# Socio-demographic and clinical factors contributing to smoking cessation among men: a four-year follow up study of the Korean Health Panel Survey

**DOI:** 10.1186/s12889-016-3583-y

**Published:** 2016-08-31

**Authors:** Joo Eun Lee, Eun-Cheol Park, Sung Youn Chun, Hye Ki Park, Tae Hyun Kim

**Affiliations:** 1 Department of Public Health, Yonsei University College of Medicine, 50 Yonsei-ro, Seodaemun-gu, Seoul, 120-752 Republic of Korea; 2 Institute of Health Services Research, Yonsei University College of Medicine, 50 Yonsei-ro, Seodaemun-gu, Seoul, 120-752 Republic of Korea; 3 Department of Preventive Medicine, Yonsei University College of Medicine, 50 Yonsei-ro, Seodaemun-gu, Seoul, 120-752 Republic of Korea; 4 Department of Hospital Administration, Graduate School of Public Health, Yonsei University, 50 Yonsei-ro,Seodaemun-gu, Seoul, 120-752 Republic of Korea

**Keywords:** Smoking cessation, Education, Marital status, Health status

## Abstract

**Background:**

To examine factors contributing to smoking cessation among male smokers, we looked at how socio-demographic and clinical characteristics influence stopping smoking with passage of time.

**Methods:**

Data from the Korea Health Panel during 2009–2012 were used. In 2009 a total of 2,941 smokers were followed up until 2012. Statistical analysis using a generalized linear mixed model was performed for all smokers, and a subgroup analysis was also performed to determine whether individual characteristics influence smoking cessation differently based on health condition.

**Results:**

Male smokers who have married or graduated college or above were more likely to succeed in smoking cessation. Those with chronic disease(s) were also more likely to quit smoking than those without. Among those without chronic disease, higher education showed significant association with smoking cessation, however, being married or ever married showed significant association with smoking cessation among those with chronic disease.

**Conclusions:**

The finding that higher education helped smokers without chronic disease succeed in smoking cessation suggests that a smoking cessation campaign should focus on those with lower education. In addition, quit smoking programs may be particularly helpful for male smokers with chronic disease(s) who have never married.

## Background

Nearly 6 million people die prematurely from tobacco use every year, including those who are exposed to second-hand smoke [1]. Smoking is the greatest risk factor to health in Organization for Economic Co-operation and Development (OECD) countries. Many tobacco control policies (public awareness campaign, bans on advertising, increased tobacco tax, expanded ban on smoking in public places, and counseling for smoking cessation) have been implemented with the aim of reducing smoking prevalence [2]. Although the smoking rate in South Korea decreased from 24.0 % in 2006 to 23.2 % in 2011, the male smoking rate in South Korea of 41.6 % continues to be much higher than the average of OECD countries, with 25.7 % in 2011. The gender gap in the adult population of smokers was particularly large in South Korea, one of the countries having the highest smoking prevalence in male and the lowest smoking prevalence in female. In addition, while the average smoking rates in OECD countries declined by 21 % between 2000 and 2011, the smoking rate in South Korea over the same period decreased by 11 % [3].

In 2015, the Korean government amended the 2003 National Health Enhancing Law and expanded smoke-free areas to include public places such as restaurants, cafes, and bars [4]. Furthermore, the government raised the cigarette tax in 2004 and 2015. Despite these efforts to reduce the smoking rate, South Korea’s anti-smoking policy ranked 25^th^ among 27 OECD countries [5]. In addition, the government’s policy has been criticized for achieving nothing besides increasing tax revenue and burdening low-income smokers [6]. Even though the overall smoking rates have decreased in South Korea, socioeconomic inequality in smoking remains high [7–9]. In this study, we attempted to examine indicators of socioeconomic status (SES), particularly education and other factors which affect smoking cessation in Korean men.

The perception of smoking risks and social support for smoking cessation may differ between men with chronic disease and those without chronic disease. The intention and ability to quit smoking are influenced by the knowledge, skills, and social support for doing so [10]. Although the Korean government has extended its anti-smoking policy, smoking cessation programs are mainly implemented by clinics and are not covered by National Health Insurance (NHI) [11]. Korea lacks an effective public campaign to reduce its smoking rate and disparity with regard to smoking cessation [12, 13]. The subgroup analyses on the basis of chronic disease, which may affect intention and ability to quit smoking, may help accurately identify the priority population. Using longitudinal data for 4 years, we attempted to investigate inequalities in smoking cessation success by SES.

## Methods

We used the four waves of the Korea Health Panel (2009, 2010, 2011, 2012) conducted by the National Health Insurance Service and the Korea Institute for Health and Social Affairs. The Korea Health Panel Survey is a longitudinal survey based on a representative sample of the entire nation. The original sample was selected using a 2-stage cluster. For the baseline survey conducted in 2009, 18,724 individuals from 6,243 households were surveyed by well-trained interviewers using face-to-face method. This study used current smokers in Korean men, who account for most smokers in Korea. Females may not accurately report smoking status because of cultural reason [14]. Therefore we used 2,972 subjects who were current smokers among men aged 18 and older in 2009 as a baseline. Current smokers were defined as adults who reported having smoked at least 100 cigarettes in their lifetime and now smoking every day or some days at baseline [15, 16]. The baseline was followed up with 2,609 subjects in 2010, 2,446 in 2011, and 2,255 in 2012. In total, 7,310 person-years were analyzed.

### Outcome measure: short-term success at smoking cessation

Short-term success at smoking cessation was assessed by surveying respondents who had not smoked for more than 6 months and were still not smoking [16]. This study excluded former smokers and newly added smokers in the baseline study population. Smoking status was measured by asking respondents “Have you smoked at least 100 cigarettes in your life time?” and if respondents answered “yes”, they were asked “Do you now smoke every day, some days, or not at all?” [17] Those who reported having smoked at least 100 cigarettes in your lifetime and smoking at the time of the baseline survey in 2009 but not smoking at the time of the follow up survey were classified as former smokers. Smokers who reported smoking cigarettes every day or some days and former smokers who reported not smoking at the time of the survey but having not smoked for less than 6 months were considered current smokers. Incidence of smoking cessation for more than 6 months was measured at each follow up wave [18].

### Independent variables

The primary independent variables were socio-demographic (SES and marital status) and clinical (chronic disease) factors. SES included education level, income level and employment status. Education level was divided into three categories: low (middle school or less), moderate (high school), and high (college or higher). Income level also divided into three categories. Employment status divided into two categories (yes or no). Marital status was coded as never married, married, and widowed/divorced/separated. Chronic disease included the number of chronic diseases, cancers, and circulatory system diseases. The number of chronic diseases was asked, “How many chronic diseases do you have?” In addition, the list of chronic diseases was asked, “What kind of chronic disease do you have?” and “Have you been diagnosed with the disease by a physician?” We included cancer and circulatory system diseases which are major chronic diseases in Korea [19].

Covariates used as categorical variables were age (18–44, 45–64, ≥65), health insurance (NHI, Medical aid), residential area (Metropolitan area, Others), cancers (No, Yes), circulatory system diseases (No, Yes), body mass index (BMI) (<23, ≥23), and drinking frequency (Never, Monthly or less, 2 to 4 times a month, 2 to 3 times a week, 4 or more times a week) [20]. The proposed cut-off value for BMI in Asian populations was 23 kg/m^2^ [21]. Independent variables collected at the time of each wave were used in the analysis.

### Statistical analysis

Chi-square test was used for descriptive statistics. This study was conducted using a generalized linear mixed model using smoking cessation between 2010 and 2012 as the dependent variable among smokers at the baseline in 2009. Excluding women, because the results of surveying women smokers may be underestimated, we focused on men aged 18 years and older at the time of the survey. To create a homogeneous group, we divided respondents by chronic disease and performed a subgroup analysis. SAS 9.4 was used for all analyses.

## Results

The descriptive statistics in men stratified by smoking cessation for each wave are shown in Table 1. Total number of male smokers was 2,971 at the baseline. Of the initial study sample, 2,609 individuals were followed up in 2010, 2,446 in 2011, and 2,255 in 2012. Among respondents who were followed up from 2010 to 2012, respondents who reported smoking cessation for more than 6 months were 7.2 % in 2010, 10.2 % in 2011, and 12.5 % in 2012. By age, smoking cessation was highest among men older than 65 years (8.4 % in 2009; 13.9 %, *p*-value = 0.0073 in 2010; 20.8 %, *p*-value < 0.0001 in 2012). A higher proportion of married men reported smoking cessation (7.8 %, *p*-value = 0.0379 in 2010; 10.9 %, *p*-value = 0.0706 in 2011; 13.9 %, *p*-value = 0.0008 in 2012). Those having no chronic disease had the lowest probability of smoking cessation (6.2 %, *p*-value = 0.0476 in 2010; 8.3 %, *p*-value = 0.0013 in 2011; 9.2 %, *p*-value < 0.0001 in 2012). In addition, men who were overweight or obese (BMI ≥ 23) (8.3 %, *p*-value = 0.00213 in 2010; 11.2 %, *p*-value = 0.0944 in 2011; 13.4 %, *p*-value = 0.1855 in 2012) reported a higher proportion of smoking cessation.

**Table 1:** Socio-demographic and clinical characteristics in male smokers between 2009 and 2012, by smoking cessation

	2009 (baseline)	2010				2011				2012			
	Smoking	Smoking		Smoking cessation		Smoking		Smoking cessation		Smoking		Smoking cessation	
	N	N	(%)	N	(%)	N	(%)	N	(%)	N	(%)	N	(%)
Age													
18–44	1,436	1,158	(93.4)	82	(6.61)	1044**	(91.6)	96**	(8.42)	942***	(90.0)	104***	(9.9)
45–64	1,134	926	(92.6)	74	(7.40)	841	(89.0)	104	(11.0)	764	(87.6)	108	(12.4)
≥65	401	338	(91.6)	31	(8.40)	311	(86.2)	50	(13.9)	267	(79.2)	70	(20.8)
Marital status													
Never married	598	466*	(95.5)	22*	(4.51)	410	(92.8)	32	(7.2)	383***	(93.0)	29***	(7.04)
Married	2,216	1,830	(92.2)	155	(7.81)	1,671	(89.1)	205	(10.9)	1,486	(86.1)	239	(13.9)
Widowed/ divorced/ separated	157	126	(92.7)	10	(7.35)	115	(89.8)	13	(10.2)	104	(88.1)	14	(11.9)
Health insurance type													
NHI	2,868	2,334	(92.7)	184	(7.31)	2,111	(89.6)	245	(10.4)	1,898	(87.4)	273	(12.6)
Medical aid	103	88	(96.7)	3	(3.30)	85	(94.4)	5	(5.6)	75	(89.3)	9	(10.7)
Residential area													
Metropolitan area	1,329	1,064	(92.6)	85	(7.40)	968	(89.7)	111	(10.3)	869	(88.3)	115	(11.7)
Others	1,642	1,358	(93.0)	102	(6.99)	1,228	(89.8)	139	(10.2)	1,104	(86.9)	167	(13.1)
Education													
≤ Middle school	675	565	(93.2)	41	(6.77)	522	(89.4)	62	(10.6)	459**	(83.5)	91**	(16.6)
High school	1,146	951	(93.5)	66	(6.49)	865	(91.1)	85	(9.0)	778	(89.2)	94	(10.8)
≥ College	1,150	906	(91.9)	80	(8.11)	809	(88.7)	103	(11.3)	736	(88.4)	97	(11.6)
Income level													
Low	982	830	(94.4)	49	(5.57)	756	(90.8)	77	(9.2)	687	(88.2)	92	(11.8)
Middle	1,010	813	(92.6)	65	(7.40)	739	(89.7)	85	(10.3)	675	(88.7)	86	(11.3)
High	979	779	(91.4)	73	(8.57)	701	(88.9)	88	(11.2)	611	(85.5)	104	(14.6)
Job													
No	540	438	(94.0)	28	(6.01)	399	(89.7)	46	(10.3)	360	(87.4)	52	(12.6)
Yes	2,431	1,984	(92.6)	159	(7.42)	1,797	(89.8)	204	(10.2)	1,613	(87.5)	230	(12.5)
Number of chronic disease													
0	1,724	1397*	(93.8)	92*	(6.18)	1259**	(91.7)	114**	(8.30)	1144***	(90.8)	116***	(9.2)
1 ~ 2	923	758	(91.0)	75	(9.00)	692	(87.8)	96	(12.2)	621	(85.0)	110	(15.1)
≥3	324	267	(93.0)	20	(6.97)	245	(86.0)	40	(14.0)	208	(78.8)	56	(21.2)
Cancers													
No	2,953	2,408	(92.9)	185	(7.13)	2,186	(89.9)	246	(10.1)	1,962	(87.6)	279	(12.5)
Yes	18	14	(87.5)	2	(12.50)	10	(71.4)	4	(28.6)	11	(78.6)	3	(21.4)
Disease of the circulatory system													
No	2,617	2,131	(92.8)	165	(7.19)	1,935	(90.1)	213	(9.9)	1,738	(88.0)	237	(12.0)
Yes	354	291	(93.0)	22	(7.03)	261	(87.6)	37	(12.4)	235	(83.9)	45	(16.1)
BMI													
<23	1,392	1138*	(94.1)	71*	(5.87)	1,027	(90.9)	103	(9.1)	930	(88.5)	121	(11.5)
≥23	1,579	1,284	(91.7)	116	(8.29)	1,169	(88.8)	147	(11.2)	1,043	(86.6)	161	(13.4)
Drinking frequency													
Never	347	278	(93.6)	19	(6.40)	244	(86.2)	39	(13.8)	212**	(81.9)	47**	(18.2)
Monthly or less	439	371	(95.9)	16	(4.13)	325	(90.8)	33	(9.2)	309	(90.1)	34	(9.9)
2 to 4 times a month	840	664	(91.7)	60	(8.3)	597	(88.7)	76	(11.3)	525	(86.0)	86	(14.0)
2 to 3 times a week	916	752	(92.3)	63	(7.7)	701	(91.5)	65	(8.5)	630	(89.9)	71	(10.1)
4 or more times a week	429	357	(92.5)	29	(7.5)	329	(89.9)	37	(10.1)	297	(87.1)	44	(12.9)
Total	2,971	2,422	(92.8)	187	(7.17)	2,196	(89.8)	250	(10.2)	1,973	(87.5)	282	(12.5)

*p < 0.001, ** *p* < 0.01, ***p < 0.05

*P*-values are for chi-square tests

Results of a generalized linear mixed model for smoking cessation in men are shown in Table 2. After adjusting for possible confounders, the table shows the association between marital status and smoking cessation. Smoking cessation was significantly higher for married men than for single men (OR = 1.994, 95 % CI = 1.407–2.828). Men with college or above were more likely to stop smoking than those with middle school or below (OR = 1.332, 95 % CI = 1.002–1.904). Higher income group also showed higher smoking cessation, but not significant (OR = 1.039, 95 % CI = 0.805–1.340 in men with middle income; OR = 1.144, 95 % CI = 0.864–1.515 in men with high income).

**Table 2:** A generalized linear mixed model: association between participant’s characteristics and smoking cessation in men

	Smoking cessation		
	OR	95 % CI	
Age (years)			
18–44	1.000		
45–64	1.058	0.799	1.399
≥65	1.422	0.937	2.159
Marital status			
Never married	1.000		
Married	1.994	1.407	2.828
Widowed/ divorced/ separated	1.436	0.800	2.578
Health insurance type			
NHI	1.000		
Medical aid	0.822	0.458	1.473
Residential area			
Metropolitan area	1.000		
Others	0.978	0.787	1.214
Education			
≤ Middle school	1.000		
High school	1.124	0.811	1.558
≥ College	1.332	1.002	1.904
Income level			
Low	1.000		
Middle	1.039	0.805	1.340
High	1.144	0.864	1.515
Job			
No	1.000		
Yes	1.024	0.776	1.352
The number of chronic diseases			
0	1.000		
1 ~ 2	1.662	1.287	2.147
≥3	2.257	1.616	3.154
Cancers			
No	1.000		
Yes	1.200	0.478	3.012
Circulatory system diseases			
No	1.000		
Yes	0.942	0.665	1.335
BMI			
<23	1.000		
≥23	1.529	1.237	1.890
Drinking frequency			
Never	1.000		
Monthly or less	1.108	0.794	1.545
2 to 4 times a month	840	0.690	1.312
2 to 3 times a week	0.664	0.475	0.927
4 or more times a week	0.499	0.338	0.738
Year			
2010	0.564	0.458	0.694
2011	0.781	0.642	0.950
2012	1.000		

The number of chronic diseases showed a significant positive association with smoking cessation (OR = 1.662, 95 % CI = 1.287–2.147 in men with 1 ~ 2 chronic diseases; OR = 2.257, 95 % CI = 1.616–3.154 in men with 3 or more chronic diseases). The table also shows the association between men who are overweight or obese and smoking cessation (OR = 1.529, 95 % CI = 1.237–1.890). Men who drink alcohol 2 or more times a week were less likely to stop smoking than those who were not drinking (OR = 0.664, 95 % CI = 0.475–0.927 in 2 to 3 times a week; OR = 0.499, 95 % CI = 0.338–0.738 in 4 or more times a week).

Results of a generalized linear mixed model stratified by chronic disease are shown in Table 3. In men without chronic disease, those older than 65 years were more likely to quit smoking cigarettes than those aged 18–44 years (OR = 2.754, 95 % CI = 1.185–6.406). Smoking cessation in men without chronic disease was significantly higher for men with high school or above than for those with middle school or below (OR = 2.133, 95 % CI = 1.006–4.520 in men with high school; OR = 2.187, 95 % CI = 1.005–4.763 in men with college or above). Men who were overweight or obese (BMI ≥ 23) were more likely to stop smoking than those with normal weight (OR = 1.597, 95 % CI = 1.161–2.196).

**Table 3:** A generalized linear mixed model: association between participant’s characteristics and smoking cessation by chronic disease in men

	Smoking cessation					
	Having no chronic disease			Having chronic disease		
	OR	95 % CI		OR	95 % CI	
Age (years)						
18–44	1.000			1.000		
45–64	1.244	0.845	1.832	0.964	0.650	1.429
≥65	2.754	1.184	6.406	1.223	0.740	2.002
Marital status						
Never married	1.000			1.000		
Married	1.688	1.080	2.576	2.963	1.571	5.59 0
Widowed/ divorced/ separated	0.611	0.176	2.125	2.523	1.124	5.665
Health insurance type						
NHI	1.000			1.000		
Medical aid	0.443	0.051	3.867	0.908	0.487	1.693
Residential area						
Metropolitan area	1.000			1.000		
Others	0.938	0.682	1.288	1.004	0.752	1.340
Education						
≤ Middle school	1.000			1.000		
High school	2.133	1.006	4.520	0.923	0.635	1.341
≥ College	2.187	1.005	4.763	1.243	0.810	1.907
Income level						
Low	1.000			1.000		
Middle	0.911	0.605	1.373	1.136	0.823	1.568
High	1.178	0.775	1.789	1.082	0.743	1.577
Job						
No	1.000			1.000		
Yes	1.262	0.788	2.002	0.898	0.640	1.262
BMI						
<23	1.000			1.000		
≥23	1.597	1.161	2.196	1.525	1.154	2.016
Drinking frequency						
Never	1.000			1.000		
Monthly or less	0.971	0.558	1.689	1.192	0.787	1.804
2 to 4 times a month	0.934	0.552	1.581	0.846	0.562	1.27 4
2 to 3 times a week	0.455	0.259	0.800	0.795	0.529	1.196
4 or more times a week	0.381	0.189	0.766	0.537	0.337	0.856
Year						
2010	0.812	0.588	1.120	0.435	0.330	0.572
2011	0.901	0.653	1.242	0.713	0.556	0.913
2012	1.000			1.000		

In terms of men with chronic disease, smoking cessation increased significantly between 2010 and 2012. In addition, smoking cessation in men with chronic disease was significantly higher for those who were married or widowed/divorced/separated than those who were never married (OR = 2.963, 95 % CI = 1.571–5.590 in married men; OR = 2.523, 95 % CI = 1.124–5.665). The table also shows that men who were overweight or obese (BMI ≥ 23) were more likely to quit smoking than those with normal weight (OR = 1.525, 95 % CI = 1.154–2.016).

## Discussion

The objective of this study was to investigate the socio-demographic and clinical factors contributing to smoking cessation among male smokers in Korean society. We found that in general, among Korean male smokers, marital status, educational level, and chronic disease were associated with success in smoking cessation. However, income differences in smoking cessation were not found in our study. In terms of men without chronic disease, among overall male smokers, men with low education attainment showed a lower rate of smoking cessation than those with high education attainment. However, among men with chronic disease, inequality in smoking cessation success showed significant association with marital status, not with educational level.

Because of cultural differences in smoking, government policies, and the gender gap, the factors contributing to smoking cessation among Korean male smokers are different from those contributing to smoking cessation other counties. In Korea, there is an old saying. “Never be friends with a person who has quit smoking,” which implies that successful ex-smokers are so bitter that it is hard to get along with them. Thus, Korean male smokers undoubtedly have some negative views on smoking cessation. Furthermore, the Korean government has a complicated relationship with the tobacco industry. The government control the profits from cigarette sales which are transferred to the public purse, but at the same time, the government’s public role is to improve public health and it also has a social responsibility to do the same [12]. The government’s policy has been criticized for filling the tax deficit with the extra tobacco tax and achieving nothing besides increasing tax revenue [6]. In addition, the gender gap in smoking rates is particularly pronounced in Korea [3]. Therefore, well-studied factors relevant to Korean society are necessary to effectively reduce its smoking rates and the disparity in smoking cessation.

A low educational level is a well-known factor associated with smoking [22–24]. With regard to education, this study showed that a high level of education was related to success in quitting smoking among Korean male smokers. In addition, smoking cessation is a preventive approach rather than a treatment for men without chronic disease, thus education is an important factor, particularly for them.

Marital status was an important factor in smoking cessation among Korean men, especially for those with chronic disease [25]. Smoking cessation showed a positive association with being both married and divorced/widowed/separated [26]. This result might indicate that in Korea, married men with chronic disease receive more social support from their spouse or other family members than never married men with chronic disease in Korea. Korean society has a unique medical culture, wherein most caregivers of patients are family members. Therefore, family support may greatly enhance smoking cessation, especially in the Korean family culture. In addition, in Korea, smoking cessation programs are primarily implemented by clinics and are not free of charge. Therefore, men with chronic disease may have more opportunities to receive advice from health professionals than those without chronic disease even those with a low educational level. Therefore, in Korean society, in the case of men with chronic disease, marital status could be a better predictor of smoking cessation than education.

In the current study, smoking cessation success in Korean male smokers was significantly associated with educational level but not with household income. Many studies conducted in the United States and Europe have shown the association between smoking cessation and income [27, 28]. Contrary to these results, some studies have shown that inequalities in smoking were clearer with education than current income [29, 30]. Because of cultural differences regarding smoking, tobacco control policies, and inequality in smoking rates, the results of these studies are difficult to generalize to other East Asian countries. Another possible explanation for the inequality in smoking cessation is the Korean government’s 2004 tobacco price increase enforcement [31, 32]. According to recent studies, effect on inequalities can differ depending on the type of tobacco control policies [33]. Tobacco price increase was likely to reduce smoking among low-income people [34]. Therefore, raising tobacco taxes had mainly a positive equity impact which reduced inequalities [35].

There are several limitations associated with this study. First, smoking cessation was measured by self-reported data without biological validation. The limitation of this survey method may result in under-reporting of smoking. Second, this study has the difficulty in generalising the results beyond South Korean male smokers. Because this study included only male smokers aged 18 and older at the baseline, differences could not be determined according to gender. Self-reported smoking prevalence in women was too low to analyze, thus women were not included in this study. Third, the data used in this study provide no information on nicotine dependence, sophisticated measure of smoking behavior, and adherence to smoking cessation treatment. However, it includes duration of smoking cessation, which makes measurement of success of smoking cessation possible. Fourth, although we used panel data, we suggest the need for caution in interpreting the results of this study. The analysis proves the association but not causality.

However, this study has several strengths. A more refined measure of smoking cessation was used in this study compared with the previous studies, with questions on never/current/former smoking and length of time since smoking cessation. Most studies defined smoking cessation without the duration of smoking cessation [26, 36]. In addition, two potentially separate stages are included in the process of smoking cessation. The important factors of quit attempt and success in quit attempt may be different. This study focused on factors related to success in smoking cessation. However, in most previous studies these two stages were not separated. Another strength of this study relates to its relatively large sample size and the longitudinal nature of the data providing a good indicator of successful smoking cessation. In addition, excluding respondents who had already quit smoking in the baseline, we tried to control confounders that had existed before the observation. Finally, we used a refined measure of chronic disease as the number of chronic diseases, prevalence of cancers, and prevalence of circulatory system diseases. Many studies on the effects of smoking cessation on chronic disease have been reported. However, in most studies on the association between smoking cessation and SES, chronic disease was not controlled [18, 37, 38].

## Conclusion

The results of this study showed that there are differences in possibility of smoking cessation success according to marital status, educational level, and chronic disease. The number of chronic diseases influences immediate health needs for treatment, so that smoking cessation can differ between men with chronic disease and those without chronic disease. After stratifying for chronic disease, educational level was a crucial factor of smoking cessation, which is a preventive approach for men without chronic disease. However, among men with chronic disease, marital status was an important social support for smoking cessation that is needed immediately for treatment of chronic disease. However, more studies on the differences of the impact of education and marital status by chronic disease are needed. In addition, differences in smoking cessation may add to inequality in smoking rate. Therefore, we suggest that effective intervention for the disadvantaged group (unmarried smokers and low educated smokers) should be a priority of tobacco control policies to improve smoking cessation.

## Abbreviations

BMI: Body mass index
NHI: National Health Insurance
OECD: Organization for Economic Co-operation and Development
SES: Socioeconomic status
